# Euthanasia of a person with a psychiatric disorder does not violate the European Convention on Human Rights (*Mortier v. Belgium* [no. 78017/17])

**DOI:** 10.1192/j.eurpsy.2022.2342

**Published:** 2022-11-10

**Authors:** M. De Hert, S. Loos, K. Van Assche

**Affiliations:** 1 Department of psychotic disorders, University Psychiatric Centre Katholieke Universiteit Leuven, Kortenberg, Belgium; 2 Department of Neurosciences, Centre for Clinical Psychiatry, Katholieke Universiteit Leuven, Leuven, Belgium; 3 Leuven Brain Institute, Katholieke Universiteit Leuven, Leuven, Belgium; 4 Antwerp Health Law and Ethics Chair, University of Antwerp, Antwerp, Belgium; 5 Research Group Personal Rights and Property Rights, Faculty of Law, University of Antwerp, Antwerp, Belgium; 6 Leuven Institute for Healthcare Policy, Katholieke Universiteit Leuven, Leuven, Belgium

**Keywords:** MAID, Euthanasia, Medical Law, Medical ethics

## Abstract

For more than 20 years, euthanasia in Belgium and The Netherlands is allowed for unbearable suffering caused by terminal or non-terminal illnesses, including psychiatric disorders. Although euthanasia numbers have been increasing over the years, the percentage of cases involving people with a primary psychiatric diagnosis has remained stable (between 1 and 2%). For these cases, the Belgian and Dutch Euthanasia Laws operate similar due care criteria: a well-considered, repeated, and voluntary request from a legally competent adult; a medical condition without prospect of improvement; constant and unbearable suffering that cannot be alleviated; consultation of two independent physicians, including a psychiatrist; and *a posteriori* evaluation and control [1–3].

For more than 20 years, euthanasia in Belgium and The Netherlands is allowed for unbearable suffering caused by terminal or non-terminal illnesses, including psychiatric disorders. Although euthanasia numbers have been increasing over the years, the percentage of cases involving people with a primary psychiatric diagnosis has remained stable (between 1 and 2%). For these cases, the Belgian and Dutch Euthanasia Laws operate similar due care criteria: a well-considered, repeated, and voluntary request from a legally competent adult; a medical condition without prospect of improvement; constant and unbearable suffering that cannot be alleviated; consultation of two independent physicians, including a psychiatrist; and *a posteriori* evaluation and control [1–3].

In the present case, the patient was a 64-year-old woman who had been suffering from chronic depression since adolescence and had also been diagnosed with a personality disorder. For many years, she received outpatient treatment by a psychiatrist. Although different therapeutic options were tried and failed, he did not want to be involved in a euthanasia request. In September 2011, she contacted Prof D. with a request for euthanasia. Prof D. and two independent psychiatrists confirmed that she was competent, experienced unbearable suffering that could not be alleviated and that was caused by a personality disorder and chronic treatment-resistant depression. During the evaluation period, the patient refused any contact with her children. In April 2012, she was euthanized by Prof D., who was (and still is) the co-chair of the Federal Control and Evaluation Commission for Euthanasia (FCECE). In June 2012, the Commission, after having examined the registration form, found that all due care criteria were met [4].

Having learned about his mother’s euthanasia, the patient’s son made several unsuccessful requests to obtain a copy of her medical file and the registration form. In April 2014, he filed a complaint against Prof D. about the euthanasia of his mother with the public prosecutor as well as the Belgian Medical Council. In 2017, the prosecutor dismissed the complaint due to a lack of evidence. In 2019, the case was reopened and a medical expert was appointed, who found that the due care criteria had been met, after which the criminal procedure was stopped. Subsequently, the patient’s son filed an application before the European Court of Human Rights (ECtHR), alleging the violation of Articles 2 and 8 of the European Convention on Human Rights (ECHR). The ECtHR is an international court ruling on the applications of individuals against a Council of Europe Member State. Its rulings have binding implications for all Member States [5].

The case of *Mortier v. Belgium* is the first ruling of the ECtHR on the compliance of euthanasia with the rights protected under the ECHR, and *a fortiori* the first ruling on euthanasia for a psychiatric disorder. In its earlier case law on end-of-life decisions (*Pretty v. the United Kingdom* 2002; *Haas v. Switzerland*, 2011; *Koch v. Germany* 2012; *Gross v. Switzerland* 2014; *Lambert and Others v. France* 2015 and 2019; *Lings v. Denmark* 2022), the ECtHR allowed Member States a wide margin of appreciation to regulate end-of-life decisions. Member States may decriminalize medical assistance in dying, but must do so in a way that guarantees the protection of the right to life (Article 2). More specifically, this will only be the case when the applicable law: (a) clearly and carefully defines the scope of the right to request medical assistance in dying; (b) provides for a procedure that can guarantee that the request is voluntary; (c) contains increased protective measures for vulnerable persons; and (d) regulates with precision the decisions that the persons tasked with assessing the request have to take to ensure the fulfillment of the due care criteria [6, 7].

The Court held that the Belgian legal framework on euthanasia for a psychiatric disorder, as outlined above, complied with these four conditions. In addition, it found that in the case of the applicant’s mother euthanasia had been performed in accordance with that legal framework. From the evidence presented, the Court was convinced that she was competent, made a well-considered, repeated, and voluntary request, and was suffering from a treatment-resistant psychiatric disorder which resulted in constant suffering that could not be alleviated. Consequently, there had been no violation of Article 2 of the ECHR.

The applicant also claimed that his right to respect for his private and family life (Article 8) had been violated, alleging that the authorities should have ensured his involvement in the process of euthanasia of his mother. In this respect, the Belgian Euthanasia Law stipulates that, if the patient so wishes, the attending physician should discuss the patient’s request with the relatives. This means that, if the patient refuses such involvement, euthanasia can rightfully be performed even without any relatives being informed. The Court indicated that the autonomy of the applicant’s mother in this regard may trump the wish of the applicant to accompany his mother in the last moments of her life. Moreover, the physicians themselves had not been allowed to contact the relatives because of their duty of confidentiality and medical secrecy. Since, in this case, the physicians had on several occasions still suggested to the applicant’s mother to resume contact with her children, to which she had objected on each occasion, the Court found that everything reasonable had been done and that Article 8 of the ECHR had not been violated.

In this regard, it should be noted that the Flemish Society of Psychiatry has recently developed guidelines for clinicians on the practice of evaluating euthanasia requests by persons with a psychiatric disorder, that contain even more stringent due care criteria than those set out in the Euthanasia Law [8]. These guidelines, formulated in response to other issues than those raised in *Mortier v. Belgium*, have in part been translated by the National Council of the Belgian Order of Physicians into deontological standards [9]. When performing euthanasia for a psychiatric disorder, physicians are now under a deontological obligation to comply with additional due care criteria: at least two of the three physicians involved should be psychiatrists; the attending physician should have face-to-face discussions with the consulted physicians about the fulfillment of all due care criteria; a patient can only be considered untreatable if all reasonable treatment options have been attempted; and the attending physician should encourage the patient to involve their relatives in the euthanasia procedure, unless there are good reasons not to do so.

Whereas in *Mortier v. Belgium*, the Court emphasized that the Belgian Euthanasia Law was human rights compliant, it still found the control system to be inadequate. The FCECE, set up to ensure the *a posteriori* control of the compliance of each case of euthanasia with the due care criteria of the Euthanasia Law, can perform its task on the basis of the anonymous part of the registration document. This, however, allows a physician who sits on the Commission and finds that he or she was involved in the euthanasia under review, to remain silent and to vote on the compatibility of his or her own action with the due care criteria of the Law. The Court emphasized that the control system had in this way failed to ensure the Commission’s independence, as it had in this case been left to the sole discretion of Prof D., the co-chair of the Commission, to recuse himself when the euthanasia that he performed was reviewed. Consequently, the Court held that there had been a violation of Article 2 of the ECHR on account of the possible lack of independence of the Commission. It should be noted that the global functioning of the FCECE and its procedures have also been questioned in recent papers [1, 10]. As a result of the ruling of the ECtHR, the Belgian legislature will be obliged to amend the *a posteriori* control procedure provided in the Euthanasia Law.

The ruling in *Mortier v. Belgium* is important because it confirms that euthanasia, including when performed on people with a psychiatric disorder, complies with human rights if the applicable Euthanasia Law offers sufficient protection against coercion and abuse. However, the ruling also calls on the Belgian legislature to revise the control system in a way that can guarantee the independence of the FCECE in each individual case. The FCECE has in the meantime issued a press release outlining its position on the implications of the judgment. According to the FCECE, only the removal of the anonymity of the registration document can remedy the shortcoming identified by the Court. We agree that the removal of anonymity is the best option to bring the control by the FCECE in line with the requirements of Article 2 ECHR. The Belgian legislature could take inspiration from the control mechanism that is established in The Netherlands. There, reporting is not anonymous, which means that each registration document always includes the identity of the physicians involved. If a physician sitting on one of the Dutch control commissions was involved in euthanasia under review, it will be obvious to all of the commission members that this person will need to recuse himself.

## References

[1] De Hert M, Loos S, Sterckx S, Thys E, Van Assche K. Improving control over euthanasia of persons with psychiatric illness: lessons from the first Belgian criminal court case concerning euthanasia. Front Psychiatry. 2022;13:933748. doi:10.3389/fpsyt.2022.933748. 35928783 PMC9343580

[2] Annual reports Regionale Toetsingscommissie (RTE). https://www.euthanasiecommissie.nl/de-toetsingscommissies/jaarverslagen; 2022 [accessed 18 October 2022].

[3] Annual reports Federal Control and Evaluation Commission for Euthanasia (FCECE). https://overlegorganen.gezondheid.belgie.be/nl/advies-en-overlegorgaan/commissies/federale-controle-en-evaluatiecommissie-euthanasie; 2022 [accessed 18 October 2022].

[4] *Mortier v. Belgium* (no. 78017/17). https://hudoc.echr.coe.int/eng?i=002-13802; 2022 [accessed 18 October 2022]

[5] Gerards J. General principles of the European Convention on Human Rights. Cambridge: Cambridge University Press; 2019.

[6] Loos S. Assisted dying before the ECtHR: general rules for national regulations. Med Law Int. 2022;22(2). doi:10.1177/0968533222107842. 92–118.

[7] ECtHR. End of life and the European Convention on Human Rights. Right to life and right to respect for private life, https://www.echr.coe.int/documents/fs_euthanasia_eng.pdf; 2022 [accessed 18 October 2022].

[8] Vlaamse Vereniging voor Psychiatrie. How to handle a patient request for euthanasia in psychiatry under current law? Advice from the Flemish Psychiatric Association (VVP) on the requirements of due care, https://www.vvp-online.be/uploads/docs/bib/euthanasie_finaal_vvp_1_dec_word_en2_docx.pdf (in Dutch); 2017 [accessed 18 October 2022].

[9] Nationale Raad van de Orde der artsen. Deontologische richtlijnen voor de toepassing van euthanasie bij patiënten die psychisch lijden ten gevolge van een psychiatrische aandoening – Actualisering advies van de nationale raad van 27 April 2019, https://ordomedic.be/nl/adviezen/ethiek/euthanasie/deontologische-richtlijnen-voor-de-toepassing-van-euthanasie-bij-pati%C3%ABnten-die-psychisch-lijden-ten-gevolge-van-een-psychiatrische-aandoening-actualisering-advies-van-de-nationale-raad-van-27-april-2019; 2022 [accessed 18 October 2022].

[10] Raus K, Vanderhaegen B, Sterckx S. Euthanasia in Belgium: shortcomings of the law and its application and of the monitoring of practice. J Med Philos. 2021;46:80–107. 33491735 10.1093/jmp/jhaa031

